# Hospital and physician-based mental healthcare during 12 months of opioid agonist treatment for opioid use disorder: Exploring costs and factors associated with acute care

**DOI:** 10.1371/journal.pone.0314296

**Published:** 2025-01-08

**Authors:** Tea Rosic, Glenda Babe, Myanca Rodrigues, Brittany B. Dennis, Richard Perez, Claire de Oliveira, Andrew Worster, Lehana Thabane, Zainab Samaan

**Affiliations:** 1 Department of Health Research Methods, Evidence and Impact, McMaster University, Hamilton, Ontario, Canada; 2 Children’s Hospital of Eastern Ontario Research Institute, Ottawa, Ontario, Canada; 3 ICES McMaster, McMaster University, Hamilton, Ontario, Canada; 4 British Columbia Centre on Substance Use, Vancouver, British Columbia, Canada; 5 Department of Medicine, McMaster University, Hamilton, Ontario, Canada; 6 Institute for Mental Health Policy Research, Centre for Addiction and Mental Health, Toronto, Ontario, Canada; 7 Campbell Family Mental Health Research Institute, Centre for Addiction and Mental Health, Toronto, Ontario, Canada; 8 ICES, Toronto, Canada; 9 Institute of Health Policy, Management and Evaluation, University of Toronto, Toronto, Canada; 10 Biostatistics Unit, Research Institute at St Joseph’s Healthcare, Hamilton, Ontario, Canada; 11 Departments of Pediatrics/Anesthesia, McMaster University, Hamilton, Ontario, Canada; 12 Faculty of Health Sciences, University of Johannesburg, Johannesburg, South Africa; 13 Department of Psychiatry and Behavioral Neurosciences, McMaster University, Hamilton, Ontario, Canada; University of Connecticut Health Center: UConn Health, UNITED STATES OF AMERICA

## Abstract

**Background:**

Individuals with opioid use disorder (OUD) have a high prevalence of co-occurring mental health disorders; however, there exists little information on mental health service use for this population. We aimed to determine the prevalence of non-substance use-related mental health emergency department (ED) visits, hospitalizations, and outpatient physician visits for individuals receiving treatment for OUD over one year. We also explored individual-level characteristics associated with mental health care service use and estimated the costs of this care.

**Methods:**

We linked observational cohort data collected from 3,430 individuals receiving treatment for OUD in Ontario, Canada, with health administrative records available for all individuals enrolled in Ontario’s public health insurance program. Eligible participants were receiving medication treatment for OUD and were recruited between 2011 and 2021 Starting on the day of cohort enrolment, we included health service data for up to 12 months. We identified ED visits and hospitalizations for non-substance use-related mental health disorders using ICD-10-CA diagnostic codes. Outpatient mental health visits to primary care providers and psychiatrists were ascertained by examining the diagnostic codes of physician billing claims. We used logistic regression to explore the association between demographic and clinical factors of interest and mental health-related ED visits or hospitalizations. Mean one-year mental healthcare costs, calculated in 2022 Canadian dollars, were estimated. We fit a two-part zero-inflated negative binomial model to explore the association between factors of interest and healthcare costs.

**Findings:**

Altogether, 14.9% of individuals had mental health-related acute care ED visits or hospitalizations and 37.3% had outpatient mental health visits during the follow up period. For participants with at least one visit, we determined the mean number of ED visits (1.93, standard deviation [SD] = 2.15), hospitalizations (1.46, SD = 1.05), primary care visits (3.51, SD = 4.31), and psychiatry visits (4.04, SD = 4.73). Lower odds of ED use and hospitalization were associated with older age (46+ compared to less than 25 years: odds ratio [OR] 0.43, 95% confidence interval [CI]: 0.29, 0.63) and being employed (OR 0.48, 95% CI 0.37, 0.61). Higher odds of ED use and hospitalization was associated with positive opioid urine drug screens (50% positive urine drug screens compared to 0%: OR 1.45, 95% CI 1.05, 2.01), having more comorbid conditions (7+ health conditions compared to 0–2 health conditions: OR 3.76, 95% CI 2.60, 5.44), and receipt of outpatient mental healthcare (OR 2.38, 95% CI 1.95, 2.92) were associated with higher odds of ED visits or hospitalizations. Mean one-year mental healthcare costs for individuals receiving ED visits or hospitalizations totaled $9,117.80 (95% CI 7,372.90, 10,862.70) per person. Mean one-year costs for individuals with outpatient mental healthcare alone totaled $382.30 (95% CI 343.20, 421.30) per person.

**Conclusions:**

Individuals receiving treatment for OUD receive care in EDs, inpatient units, and outpatient clinics for mental health conditions other than substance use-related diagnoses. Healthcare costs were considerably higher for those receiving acute care treatment for mental health conditions. Studying integrated mental health and substance use disorder treatment in the outpatient setting should be a priority to bolster care for this population.

## Introduction

Opioid use disorder (OUD) remains an unrelenting public health problem, affecting the lives of millions and causing substantial morbidity and mortality across North America [1,2]. To plan services, allocate resources, and appreciate the public health burden of the opioid crisis, efforts have been made to measure health care utilization and costs for individuals with OUD, particularly in relation to opioid misuse, poisonings, and treatment [3–5]. Individuals with OUD also experience a disproportionate number of other comorbid medical problems related to or precipitated by their illness, including infectious diseases, liver disease, cardiac disease, and pain disorders. These comorbidities, too, contribute to a high level of healthcare service utilization [6–9]. While some studies have explored patterns and predictors of healthcare service use related to medical comorbidities of OUD, there is a dearth of information on the mental health service use patterns of this population.

Many people with OUD experience other mental health disorders comorbidly. A systematic review and meta-analysis by Santo and colleagues found that among 104,135 individuals with OUD across 345 studies, the prevalence of depression was 36.1% (95% confidence interval [CI] 32.4, 39.7), and anxiety was 29.1% (95% CI 24.0, 33.3) [10]. Across Canada, more than half (56%) of hospitalizations for OUD include a co-diagnosis of another mental health disorder with sex differences observed in the prevalence and distribution of this comorbidity. Co-occurring substance use disorders are more frequently diagnosed among males than females (32% versus 23%, respectively), while mood disorders (8% versus 17%), anxiety disorders (3% versus 8%) and personality disorders (2% versus 6%) are more commonly co-diagnosed in females [11]. Mental health comorbidity is associated with negative OUD treatment outcomes [12], opioid overdoses [13], and medical complications of substance use disorders [14]. Mental health comorbidity is also associated with increased all-cause mortality and higher rates of opioid-related ED visits and hospitalizations [15].

Previous studies have focused on the impact and health system costs of medical comorbidities of patients with OUD [6–9], and patients with OUD and mental health comorbidities have been identified to have higher all-cause health system costs over 12 months than those without concurrent disorders (20539 USD vs. 10213 USD, *p* <0.01) [16]. However, exploration of health service utilization and cost specifically related to these mental health comorbidities has been missing from the literature, despite the significant burden of mental health disorders in this population. Without robust knowledge of the mental health needs of patients with OUD, it is difficult to advocate or plan for increased resources, access, and changes in healthcare delivery. Considering the prevalence and impact of comorbid mental health disorders in this population, further investigation into mental health service utilization is needed. The objective of the present study was to determine mental health service utilization for individuals with OUD during one year of opioid agonist treatment (OAT) between 2011 and 2021 in Ontario, Canada. Specifically, we aimed to first describe the prevalence of acute care (defined as emergency department (ED) visits and hospitalizations), and outpatient physician care for non-substance use-related mental health problems. We then sought to explore individual-level factors associated with acute care. Finally, we estimated costs of care and explored factors associated with healthcare costs for acute care.

## Materials and methods

**Fig 1:**
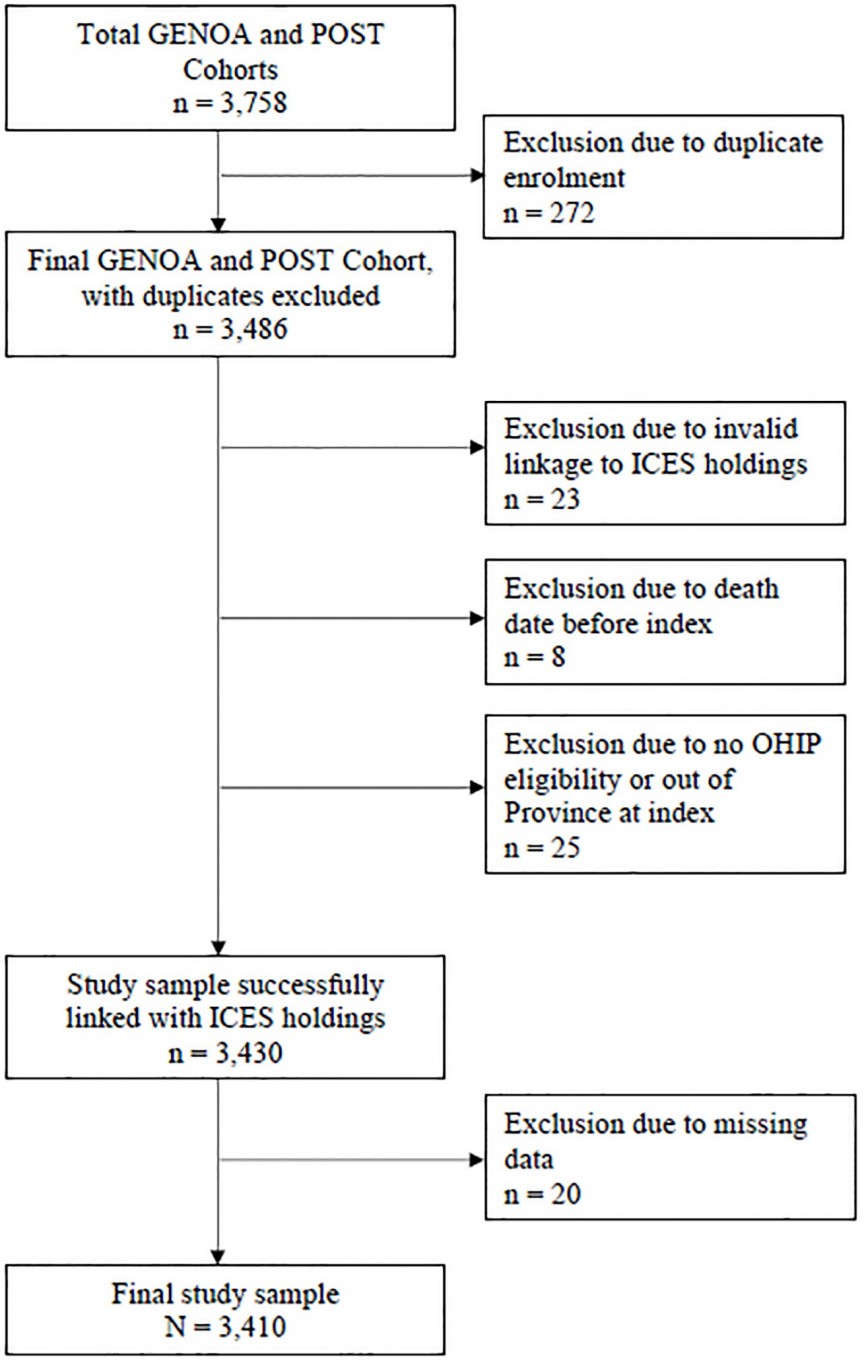
Study flow diagram.

We used data from two prospective cohort studies, the GENetics of Opioid Addiction (GENOA) study and the Pharmacogenetics of Opioid Substitution Treatment Response (POST) study. Both studies recruited individuals receiving OAT for OUD at outpatient OAT clinics across Ontario, Canada. The OAT clinics participating in this study are part of a network of clinics run centrally through the Canadian Addiction Treatment Centres. Inclusion and exclusion criteria have been previously described [17]. No exclusion criteria were applied for mental health or medical comorbidity or length of time in treatment. In the GENOA study, participants were enrolled between 2011 and 2017; in the POST study, participants were enrolled between May 2018 and April 2021. All participants provided verbal and written informed consent. Both studies were designed to study patient outcomes in OAT over 1 year and to understand biological, psychological, and social risk protective factors related to course in treatment. The POST study was an extension and expansion of the GENOA study that added several new measures, reduced the minimum age for study inclusion from 18 to 16 years of age, and expanded recruitment from 20 clinical sites to 54 clinical sites across Ontario. Both studies were conducted by the same research team using the same protocols for recruitment and data collection, allowing the cohort data to be combined without additional risks of measurement error. These studies were approved by the Hamilton Integrated Research Ethics Board (GENOA project ID 11–056; POST project ID 4556; ICES linkage project IDs 12602 and 12767-C). Data from the GENOA and POST cohort studies were merged, and duplicate enrolments excluded such that the most complete record was kept for each individual (n = 272 excluded; Fig 1).

Data were then linked with data holdings of routinely collected Ontario provincial administrative health data housed at ICES (formerly known as the Institute for Clinical Evaluative Sciences), an independent, non-profit research institute whose legal status under Ontario’s health information privacy law allows it to collect and analyze health care and demographic data, without consent, for health system evaluation and improvement. ICES holds administrative health records for all individuals enrolled in Ontario’s public health insurance program. Individual-level cohort data were linked to unique encoded identifiers at ICES through deterministic linkage using Ontario health card number and date of birth and datasets were analyzed at ICES. The following additional exclusions were made at the time of data linkage due to limitations in the linkage process: invalid linkage and lack of Ontario Health Insurance Program (OHIP) eligibility or living out of province (Fig 1). From the ICES holdings, we obtained healthcare service data for up to 12 months, starting on the day of cohort study enrolment. Follow-up was shorter than 12 months for individuals who were censored due to death (n = 39), relocation out of Province (n < 5), or due to recruitment after April 1, 2021 (n = 40) as ICES data were only available up to March 31, 2022. Individuals were not censored for discontinuing OAT. All databases were analysed at ICES, in compliance with Ontario privacy legislation. The use of these data was authorised under section 45 of Ontario’s Personal Health Information Protection Act, which does not require review by a Research Ethics Board.

We follow reporting guidelines as described in the Reporting of studies Conducted using Observational Routinely-collected Data (RECORD) and the Strengthening the Reporting of Observational Studies in Epidemiology (STROBE) guidelines [18,19].

### Mental healthcare service utilization

Data on ED visits and hospitalizations were obtained from the Canadian National Ambulatory Care Reporting System (NACRS), the Discharge Abstract Database (DAD), and the Ontario Mental Health Reporting System (OMHRS), respectively. Data from OHIP were used to identify outpatient physician visits. We aimed to exclude healthcare utilization for substance use-related disorders and thus identify only non-substance use-related mental health service utilization. This was done using International Statistical Classification of Diseases and Related Health Problems, Tenth Revision, Canada (ICD-10-CA) codes from the NACRS and DAD databases, and DSM-IV and DSM-5 codes from the OMHRS database as detailed in the ICES Mental Health and Addictions Program Framework and excluding those with a main diagnosis of a substance use-related problem [20]. This algorithm has been used in several previous ICES studies [21,22]. ED visits or hospitalizations with a main diagnosis of a substance use-related problem (ICD codes F10-19) were excluded. Outpatient physician visits were identified as mental health-related if they were: a) any visit to a psychiatrist, or b) visit to a family physician or paediatrician with an OHIP non-substance use-related mental health diagnostic code. These OHIP diagnostic codes capture visits for anxiety, depressive disorders, adjustment reaction, personality disorders, schizophrenia, bipolar disorder, sexual deviations, psychosomatic disturbances, behavioural disorders of childhood, developmental delays, autism, and dementia. We excluded OHIP mental health diagnostic codes for alcoholism, drug dependence, and tobacco use if they were the only code billed for the visit.

### Baseline characteristics

Age and sex were available from the Registered Persons Database, a population-based registry housed at ICES. Other demographic and clinical characteristics were available from the GENOA and POST cohorts, including marital status, employment status, length of time in treatment, type of OAT (methadone or buprenorphine-naloxone), mean percentage of opioid-positive drug screens and total number of opioid screens taken prior to and during study enrolment.

Using ICES holdings (i.e., DAD, OHIP, and NACRS) on co-occurring health conditions, we were able to identify the number of co-occurring health conditions (comorbidities) experienced by each participant. The number of comorbidities was assessed by applying the Canadian Institute for Health Information POP grouper algorithm to these data holdings [23]. The POP grouper algorithm uses administrative diagnosis information from inpatient and outpatient healthcare contacts to identify 226 health conditions and summarizes a profile for each individual [23]. The algorithm incorporates clinical overrides that allow redundancies to be removed after health conditions are assigned [23]. Each participant is therefore assigned a number based upon the number of unique, clinically unrelated comorbidities that are classified. Mental health and substance use disorders are included in the count of comorbidities.

### Mental healthcare cost estimation

We used a cost estimation macro, available from ICES and used in numerous previous studies to estimate mental healthcare costs related to acute care (i.e., ED visits, hospitalizations, and inpatient physician visits) and outpatient physician visits at the individual level [24–28]. In Ontario’s public healthcare system, the Ontario Ministry of Health determines the amount paid for episodes of care and individual episodes of care can therefore be assigned a cost or amount paid [26]. For mental health acute care costs, data were obtained from the NACRS, DAD, OMHRS, and OHIP billings where the location of service was inpatient or the ED. For outpatient mental healthcare costs, data were obtained from the OHIP billings claims database where the location of service was outpatient. More than 90% of all costs associated with healthcare services are captured by the ICES cost estimation algorithm [28]. We estimated mean one-year healthcare costs per person for those with ED visits only, for those with hospitalizations only, and total acute care costs (ED visits and/or hospitalizations) for those individuals with at least one ED visit or hospitalization for mental health. We estimated mean one-year outpatient healthcare costs per person for outpatient mental health visits with physicians (primary care or psychiatry) for individuals with at least one such visit. Finally, we estimated total mental healthcare costs per person (acute care and outpatient) for individuals receiving any acute care, and total mental healthcare costs for individuals receiving only outpatient mental healthcare. All costs were expressed in 2022 Canadian dollars.

### Statistical methods

We reported descriptive statistics to summarize demographic and clinical characteristics for participants with our exposure of interest, acute care measured by mental health-related ED visits and hospitalizations, and those without. We reported counts of mental health service use and costs of services using means with standard deviations. We used logistic regression to explore the association between demographic and clinical factors of interest and mental health-related acute care (ED visits and hospitalizations), reporting adjusted odds ratios (ORs) with 95% CIs. To explore the association between factors of interest and acute care mental health-related healthcare costs, we fit a two-part zero-inflated negative binomial model. The first component of the model includes a logistic regression, which estimates the risk of incurring a zero cost. The second component of the model includes a negative binomial regression to estimate the amount of healthcare costs, conditional on having a cost. This model was chosen due to excess zeros within the healthcare service use data and is commonly applied in other studies examining healthcare utilization [29–31]. This model allowed us to explore factors associated with the cost of care, accounting for having any cost in the first place. The results of this analysis are reported using ORs with 95% CI, and Cost Rate Ratios (CRR) with 95% CI, respectively. We assessed for multicollinearity by assessing variable clustering and goodness-of-fit by examining residual plots. As missing data affected just 0.6% of the total sample, multiple imputation methods were not used. All analyses were conducted using SAS Enterprise Guide 7.1 (SAS/STAT^®^ Software Version 7.1, Cary, NC, USA).

## Results

The total sample size of the merged GENOA and POST cohorts was 3,486 participants. Linkage with ICES holdings could only be accomplished for 3,430 participants (98.4% of the cohort; Fig 1). Another 20 participants were excluded due to missing data, such that the final study sample included 3,410 participants.

### Mental health service utilization

Among this cohort of individuals receiving OAT for OUD, 14.9% experienced a mental health-related ED visit or hospitalization during the study period of up to 12 months of follow up (Table 1). In Table 1, we summarize demographic and clinical characteristics for those with and without mental health-related ED visits or hospitalizations. Females accounted for 48.1% of those who received mental health-related ED visits or hospitalizations and 43.4% of those who did not. Most participants in the study cohort were between 25 and 45 years of age. Among those with mental health-related ED visits or hospitalizations, individuals younger than 25 years comprised 11%. Meanwhile, individuals younger than 25 years made up just 6.5% of the cohort without such presentations. In contrast, individuals aged 46 and older represented a greater proportion of those who did not have mental health-related ED visits or hospitalizations (27.5% versus 20.8% of those who did). Methadone was the most common type of OAT (83.8% of the total sample). Some participants were abstinent from opioid use as measured by urine drug screens (29.7% of those who had mental health-related ED visits or hospitalizations, as compared to 36.1% of those who did not), while others had urine drug screen evidence of ongoing opioid use.

**Table 1:** Demographic and clinical characteristics (N = 3,410).

Characteristic	Acute Care (ED visit/Hospitalization)	No Acute Care (ED visit/Hospitalization)
	n = 509 (14.9%)	n = 2,901 (85.1%)
**Sex**		
Female	245 (48.1%)	1,258 (43.4%)
Male	264 (51.9%)	1,643 (56.6%)
**Age (years)**		
<25	56 (11.0%)	189 (6.5%)
25–45	347 (68.2%)	1,913 (65.9%)
46+	106 (20.8%)	799 (27.5%)
**Marital status**		
Single	367 (72.1%)	2,017 (69.5%)
Married or common-law	142 (27.9%)	884 (30.5%)
**Employment status**		
Unemployed	411 (80.7%)	1,843 (63.5%)
Employed	98 (19.3%)	1,058 (36.5%)
**Type of OAT**		
Methadone	422 (82.9%)	2,436 (84.0%)
Buprenorphine-naloxone	87 (17.1%)	465 (16.0%)
**Length of time in OAT** (years)		
<2	64 (12.6%)	243 (8.4%)
2–5	66 (13.0%)	276 (9.5%)
6+	379 (74.4%)	2,382 (82.1%)
**Percentage of Opioid-positive Urine Drug Screens at study enrolment (%)**		
0	151 (29.7%)	1047 (36.1%)
1–49	275 (54.0%)	1501 (51.7%)
50+	83 (16.3%)	353 (12.2%)
**Median number of urine drug screens taken**	27 (IQR = 13–51)	34 (IQR = 14–52)
**Number of Health Conditions**		
0–2	38 (7.5%)	646 (22.3%)
3–4	92 (18.1%)	774 (26.7%)
5–6	103 (20.2%)	609 (21.0%)
7+	276 (54.2%)	872 (30.0%)
**Any Outpatient Mental Healthcare**	304 (59.7%)	969 (33.4%)
**Outpatient Primary Care Visit for Mental Health**	248 (48.7%)	803 (27.7%)
**Outpatient Psychiatry Visit for Mental Health**	165 (32.4%)	331 (11.4%)

ED = emergency department, OAT = opioid agonist treatment.

Altogether, 37.3% of individuals received at least one outpatient mental health visit with a primary care physician or psychiatrist during 12 months of OAT. Outpatient mental healthcare was received by 59.7% of individuals who experienced mental health-related ED visits or hospitalizations, meanwhile 33.4% of individuals without mental health-related ED visits or hospitalizations had any outpatient mental healthcare. Visits with primary care physicians were more common than visits with psychiatrists for both groups. Summary statistics capturing counts of mental healthcare visits are presented in Table 2. For participants who had at least one visit, the median number of ED visits was 1 (IQR = 1–2) and the median number of hospitalizations was 1 (IQR = 1–2). For participants who saw primary care physicians for mental health, the median number of visits over 12 months was 2 (IQR = 1–4). For those who saw psychiatrists, the median number of visits was 2 (IQR = 1–5).

**Table 2:** Mental health service use over 12 months for individuals with at least one acute care or outpatient mental health visit (n = 1,478).

Visit Type	Statistic
ED visit for mental health, median (IQR)	1 (1–2)
Hospitalization for mental health, median (IQR)	1 (1–2)
Outpatient primary care visit for mental health, median (IQR)	2 (1–4)
Outpatient psychiatry visit for mental health, median (IQR)	2 (1–5)

IQR = interquartile range.

### Individual-level factors associated with mental health service utilization

We identified individual-level characteristics associated with having mental health-related ED visits or hospitalizations (Table 3). Individuals aged 25–45 and those older than 46 had significantly lower odds of experiencing mental health-related ED visits or hospitalizations than individuals younger than 25 (OR 0.64, 95% CI 0.45, 0.89, and OR 0.43, 95% CI 0.29, 0.63, respectively). Individuals who were employed were similarly 50% less likely to have ED visits or hospitalizations (OR 0.48, 95% CI 0.37, 0.61). Higher levels of ongoing opioid use during treatment, as measured by more than 50% of urine drug screens positive for opioids was associated with 1.45 greater odds of mental health-related ED visits or hospitalizations (95% CI 1.05, 2.01) than abstinence from opioids. Having more health conditions was associated with increasing odds of mental health-related ED visits and hospitalizations (3–4 health conditions: OR 1.72, 95% CI 1.15, 2.56; 5–6 health conditions: OR 2.18, 95% CI 1.46, 3.24; 7+ health conditions: OR 3.76, 95% CI 2.60, 5.44). Finally, receipt of outpatient mental healthcare (primary care or psychiatry) for up to 12 months of follow up was associated with higher odds of mental health-related ED visits and hospitalizations (OR 2.38, 95% CI 1.95, 2.92).

**Table 3:** Factors associated with acute care use (N = 3,410).

Covariate	OR	95% CI	*p* value
**Sex**			
Female	[ref]		
Male	1.08	0.88, 1.32	0.482
**Age**			
<25	[ref]		
25–45	0.64	0.45, 0.89	0.009
46+	0.43	0.29, 0.63	<0.001
**Marital status**			
Single	[ref]		
Married or common law	1.07	0.86, 1.34	0.530
**Employment status**			
Unemployed	[ref]		
Employed	0.48	0.37, 0.61	<0.001
**Type of OAT**			
Methadone	[ref]		
Buprenorphine-naloxone	1.04	0.80, 1.36	0.765
**Length of time in OAT**			
<2 years	[ref]		
2–5 years	1.0	0.66, 1.51	0.989
6+ years	0.80	0.58, 1.11	0.179
**Percentage of opioid-positive urine drug screens**			
0%	[ref]		
1–49%	1.13	0.90, 1.42	0.296
50%+	1.45	1.05, 2.01	0.023
**Number of health conditions**			
0–2	[ref]		
3–4	1.72	1.15, 2.56	0.008
5–6	2.18	1.46, 3.24	<0.001
7+	3.76	2.60, 5.44	<0.001
**Outpatient Mental Healthcare (Primary care or Psychiatry)**	2.38	1.95, 2.92	<0.001

ref = reference level, OR = odds ratio, CI = confidence interval.

### Mental health-related healthcare costs

Costs estimated for mental health-related visits over 12 months are presented in Table 4. On average, ED visits cost $775.50 (95% CI 686.00, 865.10) for each individual with at least one mental health-related ED visit. The mean costs attributed to hospitalizations for individuals with at least one hospitalization were $9,103.30 (95% CI 7,181.60, 11,025.00). When acute care was considered together (ED visits and/or hospitalizations), the total mean cost estimated per individual was $7,664.80 (95% CI 6,122.30, 9,207.20) over 12 months. Outpatient mental health-related visits to primary care providers or psychiatrists cost considerably less at $441.30 (95% CI 405.50, 477.10) over 12 months for those individuals who had outpatient mental health-related visits. The total estimated mean cost of mental healthcare for individuals with any acute care visit was $9,117.80 (95% CI 7,372.90, 10,862.70) over 12 months, while the cost for individuals without any acute care was estimated at $382.30 (95% CI 343.20, 421.30).

**Table 4:** Costs in Canadian dollars of mental healthcare for those with at least one visit (n = 1,478).

Cost Type^a^	Mean (95% CI)
**Acute care**	
**ED visits-only**	775.50 (686.00, 865.10)
**Hospitalizations-only**	9,103.30 (7,181.60, 11,025.00)
**Total acute care (ED visits and/or hospitalizations)**	7,664.80 (6,122.30, 9,207.20)
**Outpatient care**	
**Outpatient physician visits**	441.30 (405.50, 477.10)
**Total Costs**	
**Total Mental Healthcare Cost**	3,250.90 (2,643.20, 3,858.60)
**Total Mental Healthcare Cost for individuals with acute care**	9,117.80 (7,372.90, 10,862.70)
**Total Mental Healthcare Cost for individuals with no acute care**	382.30 (343.20, 421.30)

^a^ For individuals with more than one visit, the cost for each visits was summed to get a total cost.

SD = standard deviation.

### Individual-level factors associated with acute care mental health-related costs

In the logistic regression component of the model, we found that age 25 and older was significantly associated with lower odds of having any mental health-related acute care costs (25–45: OR 0.64, 95% CI 0.46, 0.89; 46+: OR 0.40, 95% CI 0.27, 0.58; Table 5). Employment was associated with lower odds of having any mental health-related acute care costs compared to unemployment (OR 0.52, 95% CI 0.41, 0.65). Factors associated with higher odds of having any mental health-related acute care costs were having greater than 50% of urine drug screens positive for opioids (OR 1.40, 95% CI 1.02, 1.91), and having a greater number of health conditions (7+ health conditions: OR 3.66, 95% CI 2.61, 5.14).

**Table 5:** Two-part zero-negative binomial regression analysis to explore factors associated with total acute care mental health-related healthcare costs.

	Logistic regression component of the model^a^			Negative binomial regression component of the model^b^		
Covariate	OR	95% CI	*p* value	CRR	95% CI	*p* value
**Sex**						
Female	[ref]			[ref]		0.239
Male	1.13	0.93, 1.37	0.235	0.83	0.62, 1.13	
**Age**						
<25	[ref]			[ref]		
25–45	0.64	0.46, 0.89	0.008	1.45	0.93, 2.26	0.097
46+	0.40	0.27, 0.58	<0.001	2.01	1.19, 3.34	0.009
**Marital status**						
Single	[ref]			[ref]		
Married or common law	0.98	0.79, 1.22	0.855	0.62	0.45, 0.87	0.005
**Employment status**						
Unemployed	[ref]			[ref]		
Employed	0.52	0.41, 0.65	<0.001	0.51	0.36, 0.72	<0.001
**Type of OAT**						
Methadone	[ref]			[ref]		
Buprenorphine-naloxone	1.01	0.78, 1.31	0.940	1.28	0.86, 1.91	0.226
**Length of time in OAT**						
<2 years	[ref]			[ref]		
2–5 years	0.98	0.66, 1.46	0.925	1.01	0.59, 1.74	0.959
6+ years	0.77	0.57, 1.06	0.112	1.10	0.71, 1.70	0.675
**Percentage of opioid-positive urine drug screens**						
0%	[ref]			[ref]		
1–49%	1.16	0.93, 1.44	0.178	1.08	0.78, 1.50	0.632
50%+	1.40	1.02, 1.91	0.037	1.09	0.70, 1.71	0.705
**Number of health conditions**						
0–2	[ref]			[ref]		
3–4	1.60	1.11, 2.32	0.012	1.0	0.55, 1.81	0.988
5–6	2.00	1.38, 2.90	<0.001	0.97	0.54, 1.72	0.905
7+	3.66	2.61, 5.14	<0.001	0.93	0.56, 1.57	0.796
**Outpatient Mental Healthcare (Primary care or Psychiatry)**	2.38	1.96, 2.88	<0.001	1.47	1.09, 1.97	0.011

^a^ Probability of non-zero cost.

^b^ Expected change in cost for each unit increase in the corresponding covariate.

OR = odds ratio, RR = cost rate ratio, CI = confidence interval, ref = reference, OAT = opioid agonist treatment.

In the negative binomial component of the model, accounting for the risk of non-zero acute healthcare costs, we found that older age was associated with higher costs (CRR 2.01, 95% CI 1.19, 3.34), as was having any outpatient mental healthcare (CRR 1.47, 95% CI 1.09, 1.97). Being married or common law status was protective for acute care costs, as compared to being single (CRR 0.62, 95% CI 0.45, 0.87) as was being employed (CRR 0.51, 95% CI 0.36, 0.72).

## Discussion

Using observational cohort data linked with health administrative records, we examined mental health service utilization for individuals with OUD during one year of treatment. Nearly 15% of individuals in the cohort had ED visits or hospitalizations for mental health-related diagnoses during 12 months of follow up. These visits were not attributable to primary substance use-related diagnoses but rather represented healthcare use for other mental health comorbidities. Factors associated with receipt of acute care, in the form of ED visits and hospitalizations included younger age (less than 25 years), being unemployed, ongoing opioid use during OAT, and poorer health status (as measured by number of health conditions). We also found that 37.3% of individuals saw a primary care provider or psychiatrist for outpatient mental healthcare during OAT. Receipt of outpatient mental healthcare was associated with higher odds of ED visits or hospitalizations for mental health.

These findings shed some light on the mental health needs of individuals receiving OAT, as measured by health service use. Few prior studies have examined health service use specifically for mental health-related problems in this population. A study by Vekaria et al. found that among patients with major depressive disorder (MDD), co-occurring OUD was associated with mental health hospitalization (44% versus 36% for those with MDD only) [32]. The same was true for the use of mental health ED services (13% of those with MDD only compared to 25% of those with comorbid OUD) [32]. ICES data from Ontario has previously revealed that among nearly 56,000 individuals enrolled in OAT treatment, 87% had a diagnosis of at least one other non-substance use-related mental health disorder, with anxiety disorders (60%) and mood disorders (20%) being the most common [15]. Having OUD and a comorbid mental health disorder was associated with increased odds of death and increased incidence of ED visits or hospitalization, although reason for ED visit or hospitalization (OUD-related, medical, or mental health-related) was not explored [15].

Across Ontario, in the general population, there has been an increase in rates of visits to the ED and hospitalizations. Between 2006 and 2014, the percentage of individuals in the general population with ED visits or hospitalizations for mental health increased from 1.07% to 1.22%, with anxiety and mood disorders being the most common reasons for visit [33]. Our data demonstrate that among those with OUD, the proportion of individuals presenting for mental health-related ED visits and hospitalizations (15%) is much higher than in the general population.

The implications of needing and receiving mental health-related acute care have long been considered. The ED is frequently a first point of contact for many individuals seeking mental healthcare (for nearly half of individuals seen for incident mental health ED visits in Ontario, the visit represents their first episode of care for mental health) [34]. This is particularly true for individuals with substance use disorders or those not connected with primary care [34]. Hospitalizations for mental health disorders are typically indicated when acute safety concerns arise or when symptom severity so high that diagnostic clarification or treatment on an outpatient basis is inadequate. These acute, hospital-based services may be unavoidable for high acuity presentations, however in an optimally functioning healthcare system with timely access to outpatient mental health supports, they would ideally be avoided for lower acuity presentations.

The healthcare costs of opioid-related harms, often measured by poisonings and overdoses, have previously been studied [35–38]. However, little is known about the costs of care related to co-occurring mental health disorders in OUD. We described the 12-month costs of mental healthcare for individuals in our study cohort who received acute care and those who did not. Total costs were nearly 24 times higher for those with mental health-related ED visits and hospitalizations compared to those who received outpatient mental healthcare alone. As expected, many of the demographic and clinical factors we found to be associated with receipt of acute care mental health-related services were similarly associated with increased risk of healthcare costs, as more service use results in higher costs.

Many individuals receiving treatment for OUD do not have access to other outpatient mental health treatment. Results from a national survey conducted in the United States indicate that those with OUD and comorbid mental health disorders do not receive any mental health treatment or counseling (47% of those with mild to moderate illness and 21% of those with serious illness) [39]. For those who do receive treatment, pharmacological treatment is the most common treatment modality [39]. Another Ontario-based study revealed that for those with OUD and comorbid mental health disorders, 38% do not receive other mental health services from a primary care physician or psychiatrist in their first year of treatment [39]. Meanwhile, 39.8% have visits with a primary care physician for mental health and 12.1% have visits with both psychiatry and primary care [40].

Patients with OUD receiving treatment for their substance use disorder represent a group of individuals who may benefit from targeted health system interventions. With a high prevalence of comorbidity and numerous barriers to connection with mental health services, there is a clear need for integrated mental health and addictions care in OUD. Barriers to connection with mental health services for individuals with substance use disorders have been identified to include lack of knowledge about where to go to get treatment, fears of involuntary treatment or being forced to take medications, among others [41]. Across substance use disorders, evidence demonstrates superior outcomes for integrated treatment of substance use and mental health comorbidity, compared to siloed or sequential treatment [42]. Such compelling evidence supports the collective investment in a system of care which prioritizes integrating OUD and other mental health disorder treatment to improve access to care and outcomes [43,44]. Through a randomized controlled trial, on-site integrated addiction and mental health care has demonstrated greater initiation of mental health care, longer treatment duration, greater appointment adherence, and improvement in Global Severity Index scores than off site and non-integrated care [45]. Interventions such as contingency management improve the utilization of mental health services integrated with OAT programming [46]. A Canadian study by Morin and colleagues found that during the first year of OAT, concurrent mental health care was associated with a reduction in all-cause mortality (Risk Ratio [RR] 0.80, 95% CI 0.73, 0.87), all-cause ED visits (RR 0.87, 95% CI 0.86, 0.88), and all-cause hospitalizations (RR = 0.92, 95% CI 0.91, 0.93) [47]. There is also emerging evidence to suggest that clinical recommendations around type of OAT perhaps should be informed based upon consideration of mental health comorbidities [48], although more research is required. Hser and colleagues found that treatment with buprenorphine-naloxone led to improved opioid abstinence for those with comorbid mental health disorders, as compared to treatment with methadone (OR 0.37, 95% CI 0.21 0.66) [48]. To date, the most studied psychosocial interventions in the context of OAT are contingency management and cognitive behavioural therapy [49]. Further investigation into mental health interventions for this population is warranted and both patients and clinicians may be able to help identify interventions of interest for further study.

It is necessary to reconcile our finding that individuals who had primary care or psychiatry visits for mental health during OAT were also more likely to have ED visits and hospitalizations for mental health. Morin and colleagues somewhat similarly found that patients who received psychiatry or primary care mental health services while enrolled in OAT had higher odds of all-cause ED visits (OR 1.3, 95% CI 1.2, 1.4) but lower odds of all-cause hospitalizations (OR 0.5, 95% CI 0.4, 0.6) [40]. Whether this association reflects the higher service needs of those who do become connected with other mental healthcare while receiving OAT cannot be elucidated from the present data. At the same time, although patients frequently access OAT services (often on a daily basis), when mental health services are not integrated with addictions services, the ED may be the most direct and available route to receive care.

Finally, as is the case for the general population, we found that much of the mental healthcare for individuals with OUD is conducted by primary care physicians (as compared to psychiatrists). This speaks to the need for increased resources and training for concurrent disorders treatment in the primary care setting. Current Canadian guidelines for OUD treatment do not provide guidance on management of co-occurring mental health conditions [50]. Increased research and knowledge mobilization in this area is necessary.

### Strengths and limitations

A primary strength of this study was the ability to link observational cohort data, rich with detail about participant characteristics, to health administrative data holdings to explore service use patterns. Information on employment, marital status, and outcomes in treatment such as urine drug screen results are not available in population-level health administrative data holdings at ICES. However, this cohort of individuals receiving OAT for OUD may not be representative of the general population of individuals with OUD given their lengthy time enrolled in OAT, on average greater than 6 years. The prevalence and distribution of mental health-related health service use may be different for those with new diagnoses of OUD or those who have not been successfully connected with OUD treatment. No information was available on participants’ housing status; therefore, we were unable to assess how treatment varied for unhoused or precariously housed individuals. More research is needed to understand mental health-related health service utilization across the general population of individuals experiencing OUD. In the present study, the temporal relationship between acute care and outpatient mental health visits could not be elucidated. Given the use of observational data, we are not able to establish causation or directionality in the relationship between variables and are limited to understanding associations between factors of interest and health service outcomes. In this study, we used data harmonized from two sequentially collected cohorts therefore time effects may be present in the data collected between 2011 and 2021. An increase in treatment with buprenorphine-naloxone was observed in the POST cohort as compared to the GENOA cohort likely as a result of changing Canadian treatment guidelines, which since 2016 recommend buprenorphine-naloxone as first line medication treatment for OUD [49]. As with all administrative health records data, this studied relied on diagnostic codes, which may be affected by errors in coding or misclassification and have not been validated. In some cases, distinguishing substance use-related and non-substance use-related presentations can be a clinical challenge and therefore can present a limitation given the nature of the health administrative data and reliance on the process of diagnostic coding. It is possible that some substance use-related mental health visits failed to be classified as such due to limitations in available information about their history or (e.g., an unspecified mood disorder diagnosis in an individual who, in fact, has a substance use-related mood disorder). Additionally, we are only able to capture those cases of completed healthcare visits, and we are unable to determine how many individuals were referred to outpatient mental healthcare or whether they sought this care. Within the Canadian Addiction Treatment Centres network of OAT clinics included in this study, treatment is addiction focused and ancillary support services such as counseling services, care referrals, and community partnerships vary by location. ICES only include data on care provided by physicians, not other health care professionals such as psychologists, social workers, and nurses, who are often involved in providing mental healthcare to patients with OUD; if participants received non-physician-based mental healthcare, this would not be observed in the present data. Similarly, the costs of such non-physician-based outpatient care are not captured.

## Conclusion

Individuals with OUD have health service use including ED visits, hospitalizations, and outpatient visits for mental health conditions other than substance use-related diagnoses. Acute mental healthcare in EDs and inpatient units is higher within our cohort of patients with OUD than in the general population. Furthermore, the costs of mental health-related acute care are far higher than those for outpatient mental healthcare. This study represents a starting point for understanding mental health service use patterns for individuals with OUD and associated individual-level factors and healthcare costs. Further understanding of mental health-related health service use for individuals with new diagnoses of OUD or those who are not enrolled in treatment is needed. Likewise, research into integrating mental health and substance use disorder treatment and increasing resources within primary care where a substantial proportion of patients with concurrent disorders are seen is necessary.
